# Selection of *Pneumocystis jirovecii* Inosine 5′-Monophosphate Dehydrogenase Mutants in Solid Organ Transplant Recipients: Implication of Mycophenolic Acid

**DOI:** 10.3390/jof7100849

**Published:** 2021-10-10

**Authors:** Claire V. Hoffmann, Gilles Nevez, Marie-Christine Moal, Dorothée Quinio, Nathan Le Nan, Nicolas Papon, Jean-Philippe Bouchara, Yannick Le Meur, Solène Le Gal

**Affiliations:** 1 Laboratoire de Parasitologie et Mycologie, Hôpital de La Cavale Blanche, CHU de Brest, 29609 Brest, France; hoffmann.claire@neuf.fr (C.V.H.); dorothee.quinio@chu-brest.fr (D.Q.); 2 Groupe d’Etude des Interactions Hôte-Pathogène (GEIHP), Université d’Angers, Université de Brest, 29238 Brest, France; nathan.lenan@univ-brest.fr; 3 Département de Néphrologie, CHU de Brest, 29609 Brest, France; marie-christine.moal@chu-brest.fr (M.-C.M.); yannick.lemeur@chu-brest.fr (Y.L.M.); 4 Groupe d’Etude des Interactions Hôte-Pathogène (GEIHP), Université de Brest, Université d’Angers, 49035 Angers, France; nicolas.papon@univ-angers.fr (N.P.); jean-philippe.bouchara@univ-angers.fr (J.-P.B.); 5 UMR1227, Lymphocytes B et Autoimmunité, Université de Brest, Inserm, Labex IGO, 20609 Brest, France

**Keywords:** *Pneumocystis jirovecii*, IMPDH, transplantation, selection, mycophenolic acid

## Abstract

Mycophenolic acid (MPA) targets the inosine 5′-monophosphate dehydrogenase (IMPDH) of human lymphocytes. It is widely used as an immunosuppressant to prevent rejection in solid organ transplant (SOT) recipients who, incidentally, are at risk for *Pneumocystis* pneumonia (PCP). We hypothesized that MPA exerts selective pressure on *P. jirovecii* microorganisms considering its in vitro antifungal activity on other fungi. Thus, we analysed *impdh* gene in *P. jirovecii* isolates from SOT recipients. *P. jirovecii* specimens from 26 patients diagnosed with PCP from 2010 to 2020 were retrospectively examined: 10 SOT recipients treated with MPA and 16 non-SOT patients without prior exposure to MPA. The *P. jirovecii impdh* gene was amplified and sequenced. Nucleotide sequences were aligned with the reference sequences retrieved from available *P. jirovecii* whole genomes. The deduced IMPDH protein sequences were aligned with available IMPDH proteins from *Pneumocystis* spp. and other fungal species known to be in vitro sensitive or resistant to MPA. A total of nine SNPs was identified. One SNP (G1020A) that results in an Ala261Thr substitution was identified in all SOT recipients and in none of the non-SOT patients. Considering that IMPDHs of other fungi, resistant to MPA, harbour Thr (or Ser) at the analogous position, the Ala261Thr mutation observed in MPA-treated patients was considered to represent the signature of *P. jirovecii* exposure to MPA. These results suggest that MPA may be involved in the selection of specific *P. jirovecii* strains that circulate in the SOT recipient population.

## 1. Introduction

*Pneumocystis jirovecii* (*P. jirovecii*) is a transmissible, uncultivable fungus responsible for severe pneumonia in immunosuppressed patients. *Pneumocystis* pneumonia (PCP) remains the most frequent AIDS defining infection in HIV-infected patients in developed countries. In France, PCP represents 30% of the annually reported 1200 AIDS cases [1]. PCP also affects immunosuppressed patients not infected with HIV [2,3]. PCP occurs in 5–15% of solid organ transplant (SOT) recipients in the absence of prophylaxis, with a mortality rate up to 49% [4,5]. The frequency of PCP seems to increase in SOT recipients, specifically in renal transplant recipients (RTR), particularly when the transplantation is not recent, when the patients are no longer subjected to PCP prophylaxis and when the acquisition of *P. jirovecii* is nosocomial [6].

Mycophenolic acid (MPA) is a widely used anti-rejection treatment included in immunosuppressive regimens for SOT recipients. It targets the inosine 5′-monophosphate dehydrogenase (IMPDH), an enzyme involved in de novo purine nucleotide biosynthesis. MPA specifically inhibits the proliferation of B and T lymphocytes, since these cells synthesize de novo purines while other human cells can use alternate pathways for this synthesis [7]. Therefore, MPA is a powerful immunosuppressive drug. The resulting immunosuppression leads to opportunistic infection occurrence [8]. Nevertheless, data on the occurrence of PCP in patients treated with MPA are contradictory. MPA may have an antifungal activity on *Pneumocystis*. Oz and Hughes used a rat model which demonstrated that none of the animals treated with MPA alone or in association with dexamethasone developed PCP whereas 90% of rats treated with dexamethasone or tacrolimus alone developed PCP after 4 weeks of treatment [9]. Husain and Singh showed that PCP frequency in RTR treated with MPA was significantly lower than in RTRs without MPA treatment (0/1068 vs. 10/563, *p* = 0.00006) [8]. On the other hand, we have published three series of PCP case clusters in RTR and heart transplant recipients (HTR) and noted the occurrence of 38 PCP cases in patients effectively treated with MPA [10,11,12]. Considering these aforementioned observations, the putative antifungal activity of MPA on *P. jirovecii* remains a subject of debate. Conversely, MPA is known to have an in vitro antifungal activity on other fungal species which is useful in fundamental mycology [13].

The emergence of pathogen strains under selection pressure is widespread in microbiology and also observed for *P. jirovecii* [11,14]. In this context, the hypothesis of the emergence of particular genotypes/phenotypes of *P. jirovecii* under MPA selection pressure is relevant. The objective of the present study was to investigate this field and we retrospectively sequenced the *impdh* gene of *P. jirovecii* isolates from SOT recipients treated with MPA and a control group of non-SOT recipients with no prior exposure to MPA.

## 2. Methods

### 2.1. Hospital Settings

Brest University hospital is a 2500-bed healthcare facility, characterized by 460,000 outpatient visits and 120,000 inpatients monitored per year. Specialized care is provided for about 550 renal transplant recipients.

### 2.2. Patients and P. jirovecii Specimens

Twenty-six *P. jirovecii* specimens from 26 patients diagnosed with PCP, between 2010 and 2020, were retrospectively examined. They consisted of one group of 10 SOT recipients (T1 to T10) (sex ratio M/F 7/3; median age 65 years (55–78 years)) and another group of 16 patients who were not transplant recipients (C1 to C16) (sex ratio M/F 12/4; median age 60 years (30–89 years)). The first group had undergone MPA treatment since the day of transplantation, with a median duration of exposure of 51 months (range: 8–216 months) conversely to the second group who did not have any prior exposure to MPA (control group). Patient characteristics are summarized in Table 1 and Table 2. Biological diagnoses of PCP were initially based on microscopic detection of *P. jirovecii* and/or DNA amplification using a qPCR assay targeting the gene encoding the mitochondrial large subunit of ribosomal RNA which was performed after DNA extraction using NucliSens easyMag (bio-Mérieux, France) as we previously described [15]. *P. jirovecii* DNA specimens were stored at −80 °C for further analysis. The study was non-interventional and did not require inform consents and ethical approval according to French laws and regulations (CSP Art L1121e1.1). Specimen collection was registered with the French Ministry of Research (No. DC-2008-214).

### 2.3. P. jirovecii IMPDH Gene Analysis

The IMPDH protein of *Pneumocystis carinii* (the rat-derived *Pneumocystis* species) (NCBI accession number AAA97462.1) [16] was used as the query sequence in a BLASTp search against *P. jirovecii* proteomes at https://www.uniprot.org/blast/ (accessed on 14 September 2021). A single putative ortholog was detected in each proteome available, i.e., one ortholog (UniProt identifier A0A0W4ZU90) in the proteome from Ma and colleagues (BioProject PRJNA223510) [17] and one ortholog (UniProt identifier L0P7N3) in the proteome from Cisse and colleagues (BioProject PRJEA68827) [18]. The *P. jirovecii* gene sequences encoding the IMPDH protein were then retrieved from the European Nucleotide Archive (http://www.ebi.ac.uk/ena, accessed on 14 September 2021). The *impdh* gene corresponds to the T551_00631 locus in the *P.jirovecii* genome assembly version Pneu-jiro_RU7_V2 (BioProject PRJNA223510) [17] and to the PNEJI1_002926 locus in the *P.jirovecii* genome assembly version ASM33397v2 (BioProject PRJEA68827) [18].

Six primer pairs were designed using PrimerQuest© software (Integrated DNA Technology, https://www.idtdna.com/Primerquest/Home/Index, accessed on 14 September 2021) in order to sequence the whole *IMPDH* gene, which is 2070 bp long. Primer specificity was checked using Basic Local Alignment Search Tool (https://blast.ncbi.nlm.nih.gov/Blast.cgi, accessed on 14 September 2021). Each primer pair amplifies a DNA fragment from 376 to 915 bp in size (Table S1). Each fragment partially spanned at least one other fragment (Figure S1). The six fragments of the *impdh* gene were amplified under the same PCR conditions. PCR reactions were performed in a final volume of 50 µL containing 0.5 µM of reverse and forward primers, 3 mM MgCl_2_, 200 µM dNTPs (dNTP set, Eurogentec, Belgium) and 2 U of polymerase (HotGoldStar Diamond Taq^®^, Eurogentec, Belgium). Amplification was carried out with activation step at 95 °C for 10 min followed by 45 cycles including denaturation at 94 °C for 30 s, annealing at 60 °C for 45 s and extension at 72 °C for 1 min and a final extension step at 72 °C for 10 min. To avoid contamination, reagent preparations and amplification procedures were performed in separate rooms with different sets of micropipettes. Negative control was included in each PCR. PCR products were electrophoresed on 1.5% agarose gel containing ethidium bromide to visualize the expected band sizes (Table S1). PCR products were purified and sequenced from the two strands with the dideoxy chain termination method on the ABI 3130XL Genetic Analyzer (BigDye^®^ terminator v1.1 cycle sequencing kit, Applied Biosystem, USA). Electropherograms were analysed using PreGap and Gap software (Staden package version 2.0.0b11-2016, Staden Group). The six fragments were assembled to create the consensus sequence of the *impdh* gene from each specimen. Nucleotide and protein sequences were aligned with the reference sequences retrieved from complete *P. jirovecii* genomes as described above using BioEdit software (Version 7.0.0, Thomas Hall, Ibis Biosciences, USA) with the Clustal^®^W program. The identification of putative *IMPDH* alleles was based on the single nucleotide polymorphisms (SNPs).

Sequence logos were created using Weblogo application (https://weblogo.berkeley.edu/logo.cgi, accessed on 14 September 2021) [19].

The deduced IMPDH protein sequences of *P. jirovecii* were aligned with available IMPDH protein sequences of *Pneumocystis carinii* (Uniprot identifier A0A0W4ZEI8), *Pneumocystis murina* (Uniprot identifier M7NKD5), *Candida albicans* (Uniprot identifier Q59Q46 and GenBank accession number AAW65379.1), *Meyerozyma guilliermondii* (Uniprot identifiers A5DL50 and A5DLR4), *Saccharomyces cerevisiae* (Uniprot identifiers P38697 and P50095), *Penicillium brevicompactum* (Uniprot identifier G4WT15) and *Aspergillus nidulans* (Aspergillus Genome Database identifier AN12478) using T-coffee [20]. These protein sequences were retrieved from Uniprot, GenBank and Aspergillus Genome databases (Table S2).

### 2.4. Statistical Analysis

Frequencies of *P. jirovecii IMPDH* alleles and potential mixed infections, which were defined by the presence of more than one allele in the same specimen, were compared in the two patient groups using Fisher’s exact test. A *p* value of <0.05 was considered significant.

### 2.5. Nucleotide Sequence Accession Number

The nucleotide sequences of the *impdh* gene obtained in the present study have been deposited in GenBank under accession numbers from MZ272376 to MZ272401.

## 3. Results

A single IMPDH protein was identified within each of the two available *P. jirovecii* proteomes by a search of homology with the IMPDH protein of *P. carinii*. The two *P. jirovecii impdh* gene sequences were identical, except two synonymous SNPs at positions 1850 and 1952 (Figure S2). The *P. jirovecii impdh* gene is 2070 bp long, encompassing eight introns. The six fragments of the *impdh* gene were successfully amplified using the six designed primer pairs and afterward sequenced in all 26 patients. Consensus sequences of the *P. jirovecii impdh* gene were compared to the *impdh* gene sequence from *P. jirovecii* genome assembly version Pneu-jiro_RU7_V2 (T551_00631 locus), which was used as a reference sequence to identify SNPs [17]. Nine SNPs were identified at positions 76 (G76T), 914 (G914A), 1020 (G1020A), 1269 (C1269T), 1329 (A1329T), 1737 (G1737A), 1754 (C1754T), 1850 (T1850C) and 1952 (G1952A) (Figure S2; Figure S3; Table 3). SNP at positions 1020, 1269, 1329, 1737, 1754, 1850 and 1952 were found in at least two patients each (Table 3). SNP at positions 76 and 914 were found in only one patient each (G76T in C10 and G914A in C13) and were confirmed with a second PCR-sequencing experiment. Eight putative *IMPDH* alleles were defined based on these polymorphisms (Table 4). The sequence of the *impdh* gene from Ma and colleagues (T551_00631 locus) [17] corresponds to allele *IMPDH-1*. This allele was identified in two SOT recipients (T3 and T9) and four control patients (C1, C8, C14 and C15). The sequence of the *impdh* gene from Cissé and colleagues (PNEJI1_002926 locus) [18] corresponds to allele *IMPDH-2*. This allele was only identified in six control patients (C3, C4, C7, C9, C12 and C15). Interestingly, allele *IMPDH-3* was only identified in the 10 SOT recipients. Allele *IMPDH-4* was only identified in three control patients (C6, C14 and C16). Allele *IMPDH-5* was only identified in nine control patients (C2, C5, C7, C8, C10, C11, C12, C13, C16). Alleles *IMPDH-6*, *IMPDH-7* and *IMPDH-8* were only identified in one patient each (respectively, C2, C10 and C13).

To sum up, allele *IMPDH-3* was only identified in SOT recipients (10/10 vs. 0/16, *p* < 0.0001). Only two alleles, *IMPDH-1* and *IMPDH-3* were identified in the SOT group, whereas seven alleles, *IMPDH-1*, *IMPDH-2*, *IMPDH-4*, *IMPDH-5*, *IMPDH-6*, *IMPDH-7* and *IMPDH-8*, were identified in the control group. *IMPDH-1* was the sole common allele found in both SOT and control group. Mixed infections were observed in 2 out of 10 SOT recipients and 9 out of 16 control patients (*p* = 0.11). An overall lower diversity of alleles in SOT recipients than in control patients was observed (2 alleles in 10 patients vs. 7 alleles in 16 patients).

**Figure 1:**
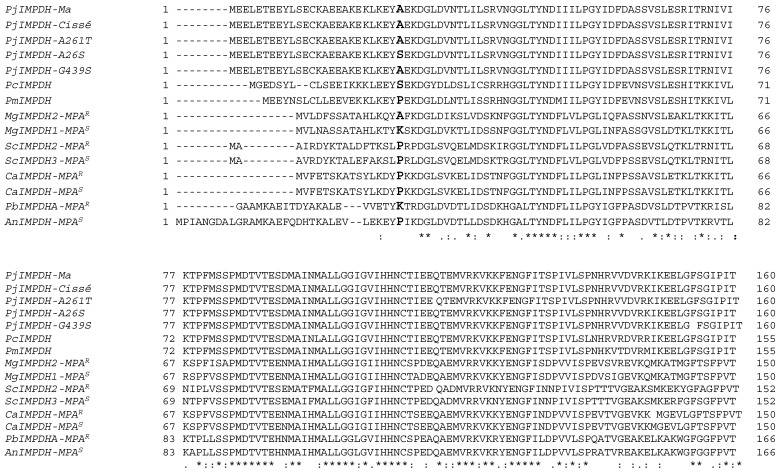

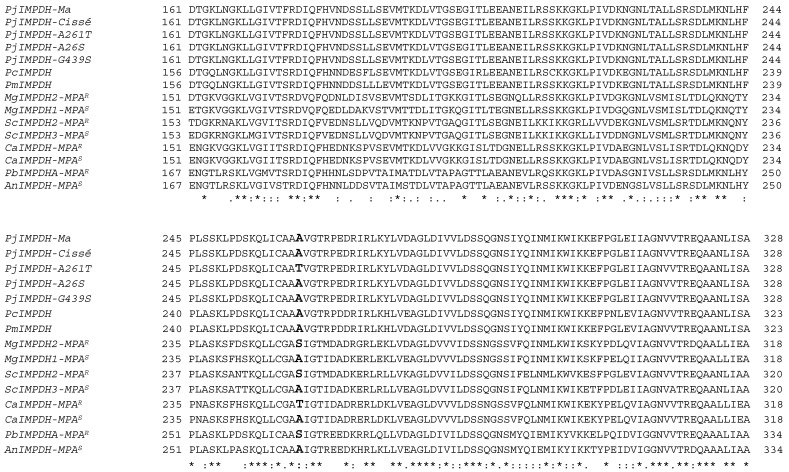

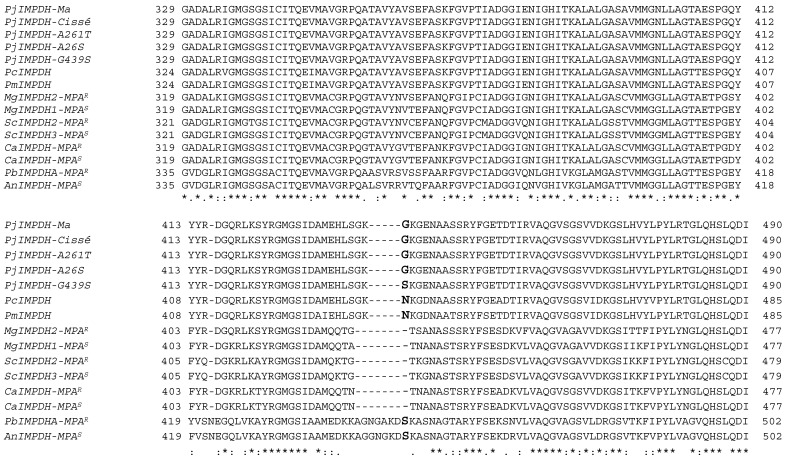

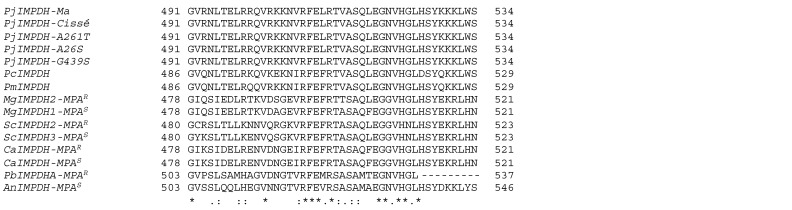
Sequence alignment of IMPDH proteins of *Pneumocystis jirovecii, Pneumocystis carinii, Pneumocystis murina, Meyerozyma guilliermondii, Saccharomyces cerevisiae, Candida albicans, Penicillium brevicompactum* and *Aspergillus nidulans*. Identical, strongly and weakly conserved residues are indicated by asterisks, double points and single points, respectively. Dashes indicate gaps. Polymorphic residues of *P. jirovecii* IMPDH and the analogous residues in other fungi are indicated in bold. *PjIMPDH-A261T*: IMPDH sequence of *P. jirovecii* with a missense mutation Ala261Thr identified in the present study; *PjIMPDH-A26S*: IMPDH sequence of *P. jirovecii* with a missense mutation Ala26Ser identified in the present study; *PjIMPDH-G439S*: IMPDH sequence of *P. jirovecii* with a missense mutation Gly439Ser identified in the present study; PjIMPDH-Ma: IMPDH sequence of *P. jirovecii* retrieved from Ma and colleagues’ proteome [17]; *PjIMPDH*-Cissé: IMPDH sequence of *P. jirovecii* retrieved from Cissé and colleagues’ proteome [18]; PcIMPDH: IMPDH sequence of *Pneumocystis carinii*; PmIMPDH: IMPDH sequence of *Pneumocystis murina*; MgIMPDH1-MPA^S^: MPA-sensitive IMPDH1 of *Meyerozyma guilliermondii*; MgIMPDH2-MPA^R^: MPA-resistant IMPDH2 of *Meyerozyma guilliermondii*; ScIMPDH2-MPA^R^: MPA-resistant IMPDH2 of *Saccharomyces cerevisiae*; ScIMPDH3-MPA^S^: MPA-sensitive IMPDH3 of *Saccharomyces cerevisiae*; CaIMPDH-MPA^R^: IMPDH of a MPA-resistant strain of *Candida albicans*; CaIMPDH-MPA^S^: MPA-sensitive IMPDH of *Candida albicans*; PbIMPDHA-MPA^R^: MPA-resistant IMPDH-A of *Penicillium brevicompactum*; AnIMPDH-MPA^S^: MPA-sensitive IMPDH of *Aspergillus nidulans*.

Consensus sequences of *P. jirovecii impdh* gene in each patient were also aligned with the coding DNA sequences (CDS) of the two reference *impdh* genes (T551_00631 and PNEJI1_002926 loci) [17,18]. SNP at positions 1269 and 1329 were located within introns (Figure S2). SNP at position 76, 914, 1020, 1737, 1754, 1850 and 1952 were located within exons. Deduced protein sequences were compared to the reference protein sequences (UniProt ID A0A0W4ZU90 and L0P7N3) [17,18]. Four synonymous mutations and 3 missense mutations were identified. Missense mutations were observed at nucleotide positions 76, 1020 and 1737. The first mutation corresponds to a transversion from G to T (G76T) and an amino acid substitution from alanine (Ala) to serine (Ser) at codon 26 (Ala26Ser) (Figure S2; Figure 1). This change characterising the allele *IMPDH-7* was found in one control patient (C10). The second mutation corresponds to a transition from G to A (G1020A) and an amino acid substitution from alanine to threonine (Thr) at codon 261 (Ala261Thr) (Figure S2; Figure 1). It is noteworthy that this change characterising the allele *IMPDH-3* was exclusively found in the SOT recipients and in all of those. The third mutation, found in three control patients (C6, C14 and C16) but not in SOT recipients, corresponds to a transition from G to A (G1737A) and an amino acid substitution from glycine to serine at codon 439 (Gly439Ser) (Figure S2; Figure 1). This change characterising the allele *IMPDH-4* was found in three control patients (C6, C14 and C16) and was not found in SOT recipients. 

*P. jirovecii* IMPDH protein sequences were aligned with available IMPDH proteins from relevant fungi, i.e., the IMPDH of other species of the *Pneumocystis* genus (*P. carinii*, *P. murina*) [17], *C. albicans* [21], *M. guilliermondii* [22], *S. cerevisiae* [23], *P. brevicompactum* [24] and *A. nidulans* [24] (Table S2) (Figure 1). The two IMPDHs of *P. jirovecii* that were retrieved from the two available proteomes (one from Ma and colleagues, the other from Cissé and colleagues) are identical and can be considered as the wild type of IMPDH [17,18]. Among the three aforementioned missense mutations identified in the present study, the mutations Ala26Ser and Gly439Ser are located within non-conserved regions (Figure 1) [24,25]. Conversely, mutation Ala261Thr is located in a strongly conserved region, Ala261 being itself a strongly conserved residue (Figure 1) [24]. Indeed, an Ala was observed at the analogous positions within the IMPDHs of *P. jirovecii* (wild type), *P. carinii* (Ala256), *P. murina* (Ala256), *C. albicans* (CaIMPDH-MPA^S^; Ala251), *A. nidulans* (Ala267) and the MPA-sensitive IMPDHs of *M. guilliermondii* (MgIMPDH1-MPA^S^; Ala251) and *S. cerevisiae* (ScIMPDH2-MPA^S^; Ala253) (Figure 1). Conversely, a Thr was observed at the analogous position within the IMPDH of an MPA-resistant *C. albicans* strain (CaIMPDH-MPA^R^; Thr251) and a Ser was observed at the analogous position within the MPA-resistant IMPDHs of *M. guilliermondii* (MgIMPDH2-MPA^R^; Ser251), *S. cerevisiae* (ScIMPDH2-MPA^R^; Ser253) and *P. brevicompactum* (Ala267).

## 4. Discussion

In this study, the first data on *P. jirovecii impdh* gene diversity were obtained. To the best of our knowledge, data on the diversity of the *impdh* gene in the most studied *Pneumocystis* species, specifically *P. carinii*, from rats and *P. murina*, from mice, are not available. Nonetheless, IMPDH of *P. carinii* as a potential drug target of MPA was previously evaluated by O’Gara and colleagues [16]. *P. carinii*’s sensitivity to MPA was established using a short-term culture of *P. carinii* [16]. This sensitivity was confirmed using an IMPDH-deficient strain of *Escherichia coli* complemented with a plasmid containing the *P. carinii impdh* gene [16].

Among the nine SNP identified in the overall *P. jirovecii* specimens from 26 patients, 6 were synonymous mutations, whereas 3 others, Ala26Ser, Ala261Thr and Gly439Ser, resulted in missense mutations. Since the two first mutations Ala26Ser and Gly439Ser are located within non-conserved regions, they might represent natural polymorphisms that might not affect protein function as it has been established in the human IMPDH1 (hIMPDH1) [7,26]. Nonetheless, since the mutation Gly439Ser is located in a hinge region, an effect on enzymatic activity cannot be ruled out.

The mutation Ala261Thr is located in a highly conserved region in fungal IMPDHs, Ala being itself a highly conserved residue, considering our alignments (Figure 1) combined with data reported by Freedman and colleagues [24] who aligned 20,000 fungal IMPDHs and found that the residues at analogous position 261 (the position identified in *P. jirovecii* IMPDH in the present study) were 91% Ala, 7.3% Gly, 1.2% Ser, 0.1% Thr and 0.1% Val [24]. Interestingly, fungi displaying natural or acquired resistance to MPA or at least naturally exposed to MPA in their environment were previously described to harbour a Ser or a Thr residue at the corresponding position in the IMPDH protein sequence. For example, a substitution from Ala to Thr (Ala251Thr) has been identified in an MPA-resistant strain of *C. albicans* selected under a high concentration of MPA (10 µg/mL) (Figure 1) [21]. Similarly, *P. brevicompactum* IMPDH-A (PbIMPDHA-MPA^R^), *S. cerevisiae* IMPDH2 (ScIMPDH2-MPA^R^) and *M. guillermondii* IMPDH2 (MgIMPDH2-MPA^R^) harbour a substitution from Ala to Ser at the analogous position (Ala267Ser, Ala253Ser and Ala251Ser, respectively) [22,23,24] (Figure 1), these three fungi being naturally resistant to MPA. Consequently, the Ala to Ser or Thr substitution in IMPDHs thus, stands out as a critical determinant of MPA-resistance in fungi [22,23,24]. Moreover, the in vitro substitution of Ala267 with Ser made the IMPDH of *Aspergillus nidulans*, a naturally MPA-sensitive fungus, resistant to MPA [24].

Interestingly, the mutation Ala261Thr in *P. jirovecii* IMPDH was found only in the 10 SOT recipients enrolled in the study, all of them being still subject to anti-rejection treatment with MPA at the date of PCP diagnosis. Nonetheless, putative resistance of *P. jirovecii* to MPA is probably not a risk factor for PCP occurrence. Indeed, the patients developed PCP because none of them were undergoing chemoprophylaxis when *P. jirovecii* infections occurred. In fact, the post-transplantation period ranged from 8 months to 216 months, whereas the duration of recommended prophylaxis is limited to 6–12 months after transplantation [5]. However, the mutation Ala261Thr among *P. jirovecii* isolates from SOT patients may represent a signature of MPA exposure and the circulation and transmission of specific *P. jirovecii* strains in susceptible patient populations. This signature of MPA exposure was previously pointed out in environmental fungal species such as *Aspergillus glaucus, Aspergillus clavatus, Aspergillus terreus,* which encode IMPDHs harbouring Ser or Thr at the analogous position [24]. These environmental moulds have probably been confronted with MPA during their evolution since they are known to develop in natural niches shared with *Penicillium* species that naturally produce MPA [24].

Consequently, MPA may have favoured the emergence of specific strains of *P. jirovecii* revealed by this specific mutation in SOT recipients. It is noteworthy that SOT recipients T3 and T9 harboured allele *IMPDH-3* (characterised by the G1020A mutation resulting in Ala261Thr) in combination with allele *IMPDH-1*, whereas the remaining eight SOT recipients harboured *IMPDH-3* solely. These mixed infections may be explained by an in-progress process of selection of *P. jirovecii* organisms under MPA pressure, wild *P. jirovecii* strains being not entirely replaced by a predominant mutant *P. jirovecii* strain. While MPA was initially considered to protect against *P. jirovecii* in the 1990s [8], an increase in PCP cases in SOT recipients has been reported, particularly in RTR, in a context of nosocomial infection over the past 15 years [6,27]. At present, MPA is identified as one of risk factors for PCP [28]. This paradigm shift could be explained in part by MPA pressure on *P. jirovecii* populations, selection of specific strains characterised by potentially lower sensitivity to MPA and acquisition/transmission of these specific strains among susceptible SOT recipients.

The phenomenon of *P. jirovecii* selection under drug pressure has already been reported with the description of specific genotypes/phenotypes of dihydropteroate synthase (DHPS) in patients treated with sulfonamides that target the DHPS [14,29,30]. Prior exposure to these drugs was identified as the main predictor of *P. jirovecii* DHPS mutants [14,29,30]. The patients’ city of residence, whether or not they had prior exposure to sulfonamides, has been identified as another predictor of *P. jirovecii* DHPS mutants [14,29]. This observation was explained by possible *P. jirovecii* transmission from infected, treated patients to susceptible, untreated patients. Similarly, we recently described a *P. jirovecii* mutant of the gene of cytochrome b (CYB), which is the target of atovaquone [11]. This mutant was spread in HTR who were effectively subject to PCP prophylaxis by atovaquone, but also in HTR without prior exposure to atovaquone, in a context of PCP outbreak [11]. For these reasons, analysis of DHPS locus and CYB genes serves as a marker of *P. jirovecii* circulation within human communities.

The presence of allele *IMPDH-3* (characterised by the G1020A mutation resulting in Ala261Thr) and the low diversity of the *P. jirovecii impdh* gene in SOT recipients suggest a common identical source of the fungus in this patient group. Conversely, the high diversity of *impdh* gene, as well as the absence of the *IMPDH-3* allele suggest several different sources of *P. jirovecii* in the control group. This diversity resulted in the identification of nine SNPs and eight alleles.

Considering the potential of *impdh* gene analysis in contributing to a method of *P. jirovecii* genotyping, we evaluated the Hunter index at 0.6952 (<0.95) [31]. This value is insufficient to distinguish *P. jirovecii* isolates in a genotyping approach based on this sole locus. Nonetheless, *impdh* gene analysis could be added to the highly discriminative multilocus sequence typing (MLST) method combining mtLSUrRNA, CYB and superoxide dismutase gene analysis, that we have used to investigate *Pneumocystis* case clusters in hospital [32]. *Impdh* gene analysis could provide an additional marker of *P. jirovecii* circulation among SOT recipients who are specifically at risk for PCP occurrence in a context of nosocomial acquisition of *P. jirovecii* and PCP outbreaks. MLST analysis including *impdh* gene may strengthen the concept of acquisition and transmission of specific *P. jirovecii* strains in SOT recipients, which has previously been discussed [6,33,34].

To sum up, we pointed out a missense mutation Ala261Thr in SOT recipients treated with MPA, whereas this change was not found in a control group without prior exposure to MPA. This mutation may represent the signature of MPA exposure and the selection of specific *P. jirovecii* strains under selection pressure that circulate in the SOT recipient population. A multicentre study, combining the enrolment of patients from diverse geographical regions and the genotyping of *P. jirovecii* specimens based on the aforementioned MLST approach is now warranted.

## Figures and Tables

**Table 1:** Characteristics of the 10 solid organ transplant recipients treated with mycophenolic acid.

Patient Code	Underlying Conditions	Post-Transplantation Period (Duration in Months) ^a^	Date of PCP Diagnosis (Year)	Examined Specimensfor PCP Diagnosis	Results of *P. jirovecii* Detection in Pulmonary Specimens Using Microscopy ^b^/PCR ^c^	PCP Treatment	Outcome
T1	RTR	73	2010	BAL	+/+	SMX-TMP	Death
T2	RTR	57	2010	BAL	+/+	SMX-TMP	Death
T3	HTR	8	2011	BAL	+/+	SMX-TMP	Recovery
T4	RTR	39	2011	BAL	+/+	SMX-TMP	Recovery
T5	RTR	33	2016	BAL	+/+	SMX-TMP ^d^ Atovaquone + corticosteroids ^e^	Death
T6	RTR	43	2017	BAL	+/+	SMX-TMP	Death
T7	RTR	151	2017	Sputum	ND/+	SMX-TMP	Recovery
T8	RTR	49	2017	BAL	+/+	SMX-TMP	Recovery
T9	RTR	53	2019	BAL	−/+	SMX-TMP	Recovery
T10	RTR + LTR	216	2020	Sputum	ND/+	SMX-TMP	Death

The male/female ratio was 7/3, the median age was 65 (range 55–78); Age and sex were not indicated in extenso, since it could be identifiable patient information; BAL: bronchoalveolar lavage; ND: not determined; +/−: positive/negative; HTR, heart transplant recipient; LTR, liver transplant recipient; RTR: renal transplant recipient; SMX-TMP: sulfamethoxazole-trimethoprim. ^a^ The post-transplantation period is also the duration of MPA exposure. ^b^ Microscopic detection of *P. jirovecii* was performed using Wright–Giemsa staining and, until December 2017, an immunofluorescence assay (MonofluoKit *P. jirovecii*, Bio-Rad). ^c^ PCR detection of *P. jirovecii* was performed using a real-time PCR assay, targeting the mitochondrial large subunit ribosomal RNA gene [15]. ^d^ First-line treatment. ^e^ Second-line treatment.

**Table 2:** Characteristics of the 16 control patients without prior exposure to mycophenolic acid.

Patient Code	Underlying Conditions	Date of PCP Diagnosis (Year)	Examined Specimensfor PCP Diagnosis	Results of *P. jirovecii* Detection in Pulmonary Specimens Using Microscopy ^a^/PCR ^b^	PCP Treatment	Outcome
C1	HM (mantle cell lymphoma)	2010	BAL	+/+	SMX-TMP	Recovery
C2	HIV	2011	BAL	+/+	SMX-TMP	Death
C3	HM (B cell lymphoma)	2012	Sputum	ND/+	Atovaquone	Death
C4	IS ^c^	2014	BAL	+/+	SMX-TMP	Recovery
C5	Cancer (glioblastoma)	2016	Sputum	ND/+	SMX-TMP	Recovery
C6	HIV	2016	BAL	+/+	SMX-TMP	Recovery
C7	HIV	2017	BAL	+/+	SMX-TMP	Recovery
C8	Cancer (bronchial carcinoma)	2018	BAL	+/+	SMX-TMP	Recovery
C9	IS ^d^	2018	BAL	+/+	SMX-TMP	Recovery
C10	HIV	2018	Sputum	ND/+	Atovaquone	Death
C11	HM (acute myeloid leukemia)	2018	BAL	-/+	SMX-TMP	Death
C12	HIV	2019	BAL	+/+	SMX-TMP	Recovery
C13	Cancer (breast adenocarcinoma)	2019	BAL	+/+	SMX-TMP	Recovery
C14	HIV	2019	BAL	+/+	SMX-TMP	Recovery
C15	Cancer (bronchial carcinoma)	2019	Sputum	ND/+	SMX-TMP	Recovery
C16	IS ^e^	2020	BAL	+/+	SMX-TMP	Recovery

The male/female ratio was 12/4, the median age was 60 (range 30–89); age and sex were not indicated in extenso since it could be identifiable patient information; BAL: bronchoalveolar lavage; ND: not determined; +/−: positive/negative; HM: haematological malignancy; HIV: human immunodeficiency virus; IS: immunosuppressive therapy; SMX-TMP: sulfamethoxazole-trimethoprim. ^a^ Microscopic detection of *P. jirovecii* was performed using Wright–Giemsa staining and, until December 2017, an immunofluorescence assay (MonofluoKit *P. jirovecii*, Bio-Rad). ^b^ PCR detection of *P. jirovecii* was performed using a real-time PCR assay, targeting the mitochondrial large subunit ribosomal RNA gene [15]. ^c^ Azathioprine, anti-TNFα and corticosteroids treatments for Crohn’ disease. ^d^ Methotrexate and corticosteroids treatments Horton’s disease. ^e^ Methotrexate treatment for psoriasis.

**Table 3:** Single nucleotide polymorphisms of *Pneumocystis jirovecii impdh* gene in 10 solid organ transplant recipients with prior exposure to mycophenolic acid and 16 control patients which were not SOT recipients and had no prior exposure to MPA.

Patient Code	Nucleotide Position (bp)									*IMPDH* Allele
	76	914	1020	1269	1329	1737	1754	1850	1952	
SOT recipients										
T1	G	G	A	C	A	G	C	T	G	*IMPDH-3*
T2	G	G	A	C	A	G	C	T	G	*IMPDH-3*
T3	G	G	A/G ^a^	C	A	G	C	T	G	*IMPDH1 + IMPDH-3*
T4	G	G	A	C	A	G	C	T	G	*IMPDH-3*
T5	G	G	A	C	A	G	C	T	G	*IMPDH-3*
T6	G	G	A	C	A	G	C	T	G	*IMPDH-3*
T7	G	G	A	C	A	G	C	T	G	*IMPDH-3*
T8	G	G	A	C	A	G	C	T	G	*IMPDH-3*
T9	G	G	A/G ^a^	C	A	G	C	T	G	*IMPDH-1 + IMPDH-3*
T10	G	G	A	C	A	G	C	T	G	*IMPDH-3*
Control patients										
C1	G	G	G	C	A	G	C	T	G	*IMPDH-1*
C2	G	G	G	C	A/T ^a^	G	C	T	G/A^a^	*IMPDH-5 + IMPDH-6*
C3	G	G	G	C	A	G	C	C	A	*IMPDH-2*
C4	G	G	G	C	A	G	C	C	A	*IMPDH-2*
C5	G	G	G	C	T	G	C	T	G	*IMPDH-5*
C6	G	G	G	T	A	A	T	T	G	*IMPDH-4*
C7	G	G	G	C	A/T ^a^	G	C	C/T ^a^	G/A ^a^	*IMPDH-2 + IMPDH-5*
C8	G	G	G	C	A/T ^a^	G	C	T	G	*IMPDH-1+ IMPDH-5*
C9	G	G	G	C	A	G	C	C	A	*IMPDH-2*
C10	G/T	G	G	C	A/T ^a^	G	C	T	G	*IMPDH-5 + IMPDH-7*
C11	G	G	G	C	T	G	C	T	G	*IMPDH-5*
C12	G	G	G	C	A/T ^a^	G	C	C/T ^a^	G/A ^a^	*IMPDH-2 + IMPDH-5*
C13	G	A/G	G	C	A/T ^a^	G	C	T	G	*IMPDH-5 + IMPDH-8*
C14	G	G	G	C/T ^a^	A	A/G ^a^	C/T ^a^	T	G	*IMPDH-1 + IMPDH-4*
C15	G	G	G	C	A	G	C	C/T ^a^	G/A ^a^	*IMPDH-1 + IMPDH-2*
C16	G	G	G	C/T ^a^	A/T ^a^	A/G ^a^	C/T ^a^	T	G	*IMPDH-4 + IMPDH-5*

^a^ Two nucleotides were identified at this position on sequence electropherograms.

**Table 4:** *Pneumocystis jirovecii IMPDH* alleles defined in the present study.

Nucleotide Positions (bp)									*IMPDH* Allele
76	914	1020	1269	1329	1737	1754	1850	1952	
G	G	G	C	A	G	C	T	G	*IMPDH-1*
G	G	G	C	A	G	C	C	A	*IMPDH-2*
G	G	A	C	A	G	C	T	G	*IMPDH-3*
G	G	G	T	A	A	T	T	G	*IMPDH-4*
G	G	G	C	T	G	C	T	G	*IMPDH-5*
G	G	G	C	A	G	C	T	A	*IMPDH-6*
T	G	G	C	A	G	C	T	G	*IMPDH-7*
G	A	G	C	A	G	C	T	G	*IMPDH-8*

## Data Availability

The nucleotide sequences of the *impdh* gene obtained in the present study have been deposited in GenBank under accession numbers from MZ272376 to MZ272401.

## Supplementary Materials

The following are available online at https://www.mdpi.com/article/10.3390/jof7100849/s1, Figure S1: Description of PCR products obtained using the six primer pairs designed for Pneumocystis jirovecii impdh gene amplification, Figure S2: Alignment of impdh CDS and genomic sequences of the two P.jirovecii genome assemblies, Figure S3: Diversity of the impdh gene of Pneumocystis jirovecii based on the analysis of 26 specimens, Table S1: Nucleotide sequences of primers used for amplification of Pneumocystis jirovecii impdh gene, Table S2: List of the IMPDH protein sequences from *Pneumocystis* species and other fungi that were aligned with the IMPDH sequences obtained in the present study.
