# Supplementary material for: Selection of Pneumocystis jirovecii Inosine 5′-Monophosphate Dehydrogenase Mutants in Solid Organ Transplant Recipients: Implication of Mycophenolic Acid
Source: J Fungi (Basel). 2021 Oct 10;7(10):849. doi: 10.3390/jof7100849 (PMC8537117; doi:10.3390/jof7100849)
Supplement: Supplementary file 1 [file jof-07-00849-s001.zip › jof-1402122-supplementary/Table S2.pdf]

Table S2. List of the IMPDH protein sequences from *Pneumocystis* species and other fungi that were aligned with the IMPDH sequences obtained in the present study.

**Organism – strain**	**Abbreviation**	**Protein sequence**	**Protein length (aa)**	**Comments**
Pneumocystis jirovecii	PjIMPDH-Ma	Uniprot A0A0W4ZU90	534	IMPDH from Ma et al. [17]
Pneumocystis jirovecii	PjIMPDH-Cissé	Uniprot L0P7N3	534	IMPDH from Cissé et al. [18]
Pneumocystis carinii	PcIMPDH	Uniprot A0A0W4ZEI8	529	IMPDH from Ma et al. [17]
Pneumocystis murina	PmIMPDH	Uniprot M7NKD5	529	IMPDH from Ma et al. [18]
Meyerozyma guilliermondii	MgIMPDH1-MPA^{S}	Uniprot A5DL50	521	MPA-sensitive IMPDH produced by *IMH3.1* gene [a]
Meyerozyma guilliermondii	MgIMPDH2-MPA^{R}	Uniprot A5DLR4	521	MPA-resistant IMPDH produced by *IMH3.2* gene [a]
***Saccharomyces cerevisiae* S288C**	ScIMPDH2-MPA^{R}	Uniprot P38697	523	MPA-resistant IMPDH produced by *IMD2* gene [b]
***Saccharomyces cerevisiae* S288C**	ScIMPDH3-MPA^{S}	Uniprot P50095	523	MPA-sensitive IMPDH produced by *IMD3* gene [b]
***Candida albicans* EC3**	CaIMPDH-MPA^{R}	GenBank AAW65379.1	521	MPA-resistant IMPDH [c]
***Candida albicans* SC5314**	CaIMPDH-MPA^{S}	Uniprot Q59Q46	521	MPA-sensitive IMPDH
Penicillium brevicompactum	PbIMPDHA-MPA^{R}	Uniprot G4WT15	537	MPA-resistant IMPDH (IMPDH-A)<br>Partial coding sequence missing N-terminal MPITASDAAP
Aspergillus nidulans	AnIMPDH-MPA^{S}	Aspergillus Genome Database AN12478	546	MPA-sensitive IMPDH

[a] *Meyerozyma guillermondii* genome harbors two genes (*IMH3.1* and *IMH3.2*) encoding IMPDH proteins. *IMH3.1* encodes a MPA-sensitive IMPDH whereas *IMH3.2* encodes a MPA-resistant IMPDH [22].

[b] *Saccharomyces cerevisiae* genome harbors four genes (*IMD1 – IMD4*) encoding IMPDH proteins. *IMD1*, *IMD3* and *IMD4* encode MPA-sensitive IMPDHs whereas *IMD2* encodes a MPA-resistant IMPDH [23].

[c] *Candida albicans* EC3 is a mutant strain of strain SC5314 selected under high concentration of MPA (10 µg/mL) [21].
