# Supplementary material for: Selection of Pneumocystis jirovecii Inosine 5′-Monophosphate Dehydrogenase Mutants in Solid Organ Transplant Recipients: Implication of Mycophenolic Acid
Source: J Fungi (Basel). 2021 Oct 10;7(10):849. doi: 10.3390/jof7100849 (PMC8537117; doi:10.3390/jof7100849)
Supplement: Supplementary file 1 [file jof-07-00849-s001.zip › jof-1402122-supplementary/Table S1.pdf]

Table S1. Nucleotide sequences of primers used for amplification of *Pneumocystis jirovecii impdh* gene

Primer pair	Primer designation	5'-3' nucleotide sequence	Amplicon size (bp)
**1**	2116F	TGGTGACAATACAATAGGTGATG	743
	2859R	CATTCGTGGCGGAAGATT	
**2**	1537F	CTAATCCCGCGTCAACTAAATA	915
	2452R	CGGGTCAATGGAGGATTAAC	
**3**	1109F	CCATCTGCAATAGTTGGTACTC	681
	1790R	AGTAACAGGATCAGAGGGTATT	
**4**	745F	GAAGCCCGGTTCGTAAATAAG	846
	1591R	GGAACACGACCTGAAGATAGA	
**5**	616F	GATAAATACCGTGGACATTCCC	376
	992R	TTTAGCTGGAACAGCAGAATC	
**6**	295F	TCCTGAAGAATCTCGCTCAATA	512
	807R	CAGGAAGTGTTGTGGATAAAGG	

F : Forward; R : Reverse
