# Supplementary material for: Selection of Pneumocystis jirovecii Inosine 5′-Monophosphate Dehydrogenase Mutants in Solid Organ Transplant Recipients: Implication of Mycophenolic Acid
Source: J Fungi (Basel). 2021 Oct 10;7(10):849. doi: 10.3390/jof7100849 (PMC8537117; doi:10.3390/jof7100849)
Supplement: Supplementary file 1 [file jof-07-00849-s001.zip › jof-1402122-supplementary/Figure S1.pdf]

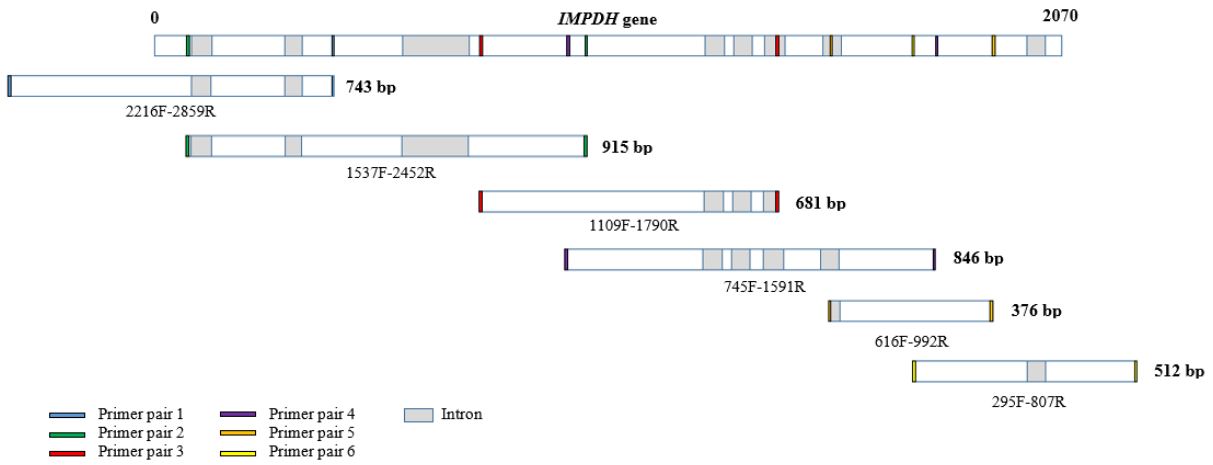

Figure S1. Description of PCR products obtained using the six primer pairs designed for *Pneumocystis jirovecii impdh* gene amplification
