# Supplementary material for: Selection of Pneumocystis jirovecii Inosine 5′-Monophosphate Dehydrogenase Mutants in Solid Organ Transplant Recipients: Implication of Mycophenolic Acid
Source: J Fungi (Basel). 2021 Oct 10;7(10):849. doi: 10.3390/jof7100849 (PMC8537117; doi:10.3390/jof7100849)
Supplement: Supplementary file 1 [file jof-07-00849-s001.zip › jof-1402122-supplementary/Figure S2.pdf]

Figure S2. Alignment of *impdh* CDS and genomic sequences of the two *P.jirovecii* genome assemblies (Ma *et al.*, 2016, indicated as Ma ; Cissé *et al.*, 2012, indicated as Cisse) [17,18]. Residues shown in bold and highlighted in grey are synonymous SNPs. Dashes indicate gaps. Nucleotide positions where SNPs were identified in the present study are indicated with asterisks.

```
Pj_impdh_Ma_genomic     ATGGAGGAATTAGAAACAGAAGAATATTTAAGTGAATGTAAAGCTGAAGAAGCAAAGGAAAAACTTAAAG 70
Pj_impdh_Ma_CDS         ATGGAGGAATTAGAAACAGAAGAATATTTAAGTGAATGTAAAGCTGAAGAAGCAAAGGAAAAACTTAAAG 70
Pj_impdh_Cisse_genomic  ATGGAGGAATTAGAAACAGAAGAATATTTAAGTGAATGTAAAGCTGAAGAAGCAAAGGAAAAACTTAAAG 70
Pj_impdh_Cisse_CDS      ATGGAGGAATTAGAAACAGAAGAATATTTAAGTGAATGTAAAGCTGAAGAAGCAAAGGAAAAACTTAAAG 70

                                *
Pj_impdh_Ma_genomic     AATATGCAGAAAAAGTATGTAAAATCAGAGTATTTTTATTGAAAATTAACAAAAATAAAAGGATGGATTA 140
Pj_impdh_Ma_CDS         AATATGCAGAAAAAG-----------------------------------------------ATGGATTA 93
Pj_impdh_Cisse_genomic  AATATGCAGAAAAAGTATGTAAAATCAGAGTATTTTTATTGAAAATTAACAAAAATAAAAGGATGGATTA 140
Pj_impdh_Cisse_CDS      AATATGCAGAAAAAG-----------------------------------------------ATGGATTA 93

Pj_impdh_Ma_genomic     GACGTGAATACTCTTATTCTTAGCCGGGTCAATGGAGGATTAACATATAATGATATAATTATTCTTCCGG 210
Pj_impdh_Ma_CDS         GACGTGAATACTCTTATTCTTAGCCGGGTCAATGGAGGATTAACATATAATGATATAATTATTCTTCCGG 163
Pj_impdh_Cisse_genomic  GACGTGAATACTCTTATTCTTAGCCGGGTCAATGGAGGATTAACATATAATGATATAATTATTCTTCCGG 210
Pj_impdh_Cisse_CDS      GACGTGAATACTCTTATTCTTAGCCGGGTCAATGGAGGATTAACATATAATGATATAATTATTCTTCCGG 163

Pj_impdh_Ma_genomic     GATATATTGATTTTGATGCAAGCTCAGTATCTCTTGAAAGTCGTATTACGAGAAATATAGTTATAAAAAC 280
Pj_impdh_Ma_CDS         GATATATTGATTTTGATGCAAGCTCAGTATCTCTTGAAAGTCGTATTACGAGAAATATAGTTATAAAAAC 233
Pj_impdh_Cisse_genomic  GATATATTGATTTTGATGCAAGCTCAGTATCTCTTGAAAGTCGTATTACGAGAAATATAGTTATAAAAAC 280
Pj_impdh_Cisse_CDS      GATATATTGATTTTGATGCAAGCTCAGTATCTCTTGAAAGTCGTATTACGAGAAATATAGTTATAAAAAC 233

Pj_impdh_Ma_genomic     GCCATTTATGAGGTTTTCATGTTATTTTTATTGCTAGAGTATTAATGTATAGTTCTCCAATGGATACTGT 350
Pj_impdh_Ma_CDS         GCCATTTATGAG---------------------------------------TTCTCCAATGGATACTGT 263
Pj_impdh_Cisse_genomic  GCCATTTATGAGGTTTTCATGTTATTTTTATTGCTAGAGTATTAATGTATAGTTCTCCAATGGATACTGT 350
Pj_impdh_Cisse_CDS      GCCATTTATGAG---------------------------------------TTCTCCAATGGATACTGT 263

Pj_impdh_Ma_genomic     TACTGAATCGGATATGGCGATTAACATGGCACTCCTAGGGGGCATTGGAGTTATTCACCATAATTGTACA 420
Pj_impdh_Ma_CDS         TACTGAATCGGATATGGCGATTAACATGGCACTCCTAGGGGGCATTGGAGTTATTCACCATAATTGTACA 333
Pj_impdh_Cisse_genomic  TACTGAATCGGATATGGCGATTAACATGGCACTCCTAGGGGGCATTGGAGTTATTCACCATAATTGTACA 420
Pj_impdh_Cisse_CDS      TACTGAATCGGATATGGCGATTAACATGGCACTCCTAGGGGGCATTGGAGTTATTCACCATAATTGTACA 333

Pj_impdh_Ma_genomic     ATAGAAGAACAGACGGAAATGGTTAGAAAAGTTAAAAAATTTGAAAATGGATTTATAACATCACCTATTG 490
Pj_impdh_Ma_CDS         ATAGAAGAACAGACGGAAATGGTTAGAAAAGTTAAAAAATTTGAAAATGGATTTATAACATCACCTATTG 403
Pj_impdh_Cisse_genomic  ATAGAAGAACAGACGGAAATGGTTAGAAAAGTTAAAAAATTTGAAAATGGATTTATAACATCACCTATTG 490
Pj_impdh_Cisse_CDS      ATAGAAGAACAGACGGAAATGGTTAGAAAAGTTAAAAAATTTGAAAATGGATTTATAACATCACCTATTG 403

Pj_impdh_Ma_genomic     TATTGTCACCAAACCACAGAGTGGTTGATGTTCGTAAAATAAAAGAAGAGCTTGGATTTAGTGGGATTCC 560
Pj_impdh_Ma_CDS         TATTGTCACCAAACCACAGAGTGGTTGATGTTCGTAAAATAAAAGAAGAGCTTGGATTTAGTGGGATTCC 473
Pj_impdh_Cisse_genomic  TATTGTCACCAAACCACAGAGTGGTTGATGTTCGTAAAATAAAAGAAGAGCTTGGATTTAGTGGGATTCC 560
Pj_impdh_Cisse_CDS      TATTGTCACCAAACCACAGAGTGGTTGATGTTCGTAAAATAAAAGAAGAGCTTGGATTTAGTGGGATTCC 473

Pj_impdh_Ma_genomic     AATAACAGGTAACATATAAAAATATAGACTGAAAATATGATGAATTTATTTTTTAAACTTTCGTTCTACC 630
Pj_impdh_Ma_CDS         AATAACAG-------------------------------------------------------------- 481
Pj_impdh_Cisse_genomic  AATAACAGGTAACATATAAAAATATAGACTGAAAATATGATGAATTTATTTTTTAAACTTTCGTTCTACC 630
Pj_impdh_Cisse_CDS      AATAACAG-------------------------------------------------------------- 481

Pj_impdh_Ma_genomic     GTATATCACTTATTAGTTGTGTGGAAACATTTATCTGATTTGGTTTAAATATATATCATTTATATGCATG 700
Pj_impdh_Ma_CDS         ---------------------------------------------------------------------- 481
Pj_impdh_Cisse_genomic  GTATATCACTTATTAGTTGTGTGGAAACATTTATCTGATTTGGTTTAAATATATATCATTTATATGCATG 700
Pj_impdh_Cisse_CDS      ---------------------------------------------------------------------- 481

Pj_impdh_Ma_genomic     GATAATAATAATGTGTCTAGATACAGGAAAACTCAACGGGAAGCTTTTGGGGATTGTTACGTTTAGAGAT 770
Pj_impdh_Ma_CDS         --------------------ATACAGGAAAACTCAACGGGAAGCTTTTGGGGATTGTTACGTTTAGAGAT 531
Pj_impdh_Cisse_genomic  GATAATAATAATGTGTCTAGATACAGGAAAACTCAACGGGAAGCTTTTGGGGATTGTTACGTTTAGAGAT 770
Pj_impdh_Cisse_CDS      --------------------ATACAGGAAAACTCAACGGGAAGCTTTTGGGGATTGTTACGTTTAGAGAT 531

Pj_impdh_Ma_genomic     ATTCAGTTTCATGTTAACGATTCTTCATTACTTTCGGAAGTCATGACAAAAGATTTAGTAACAGGATCAG 840
Pj_impdh_Ma_CDS         ATTCAGTTTCATGTTAACGATTCTTCATTACTTTCGGAAGTCATGACAAAAGATTTAGTAACAGGATCAG 601
Pj_impdh_Cisse_genomic  ATTCAGTTTCATGTTAACGATTCTTCATTACTTTCGGAAGTCATGACAAAAGATTTAGTAACAGGATCAG 840
Pj_impdh_Cisse_CDS      ATTCAGTTTCATGTTAACGATTCTTCATTACTTTCGGAAGTCATGACAAAAGATTTAGTAACAGGATCAG 601

Pj_impdh_Ma_genomic     AGGGTATTACCCTTGAAGAGGCAAATGAAATCCTCCGTTCTTCTAAAAAAGGGAAATTGCCTATAGTTGA 910
Pj_impdh_Ma_CDS         AGGGTATTACCCTTGAAGAGGCAAATGAAATCCTCCGTTCTTCTAAAAAAGGGAAATTGCCTATAGTTGA 671
Pj_impdh_Cisse_genomic  AGGGTATTACCCTTGAAGAGGCAAATGAAATCCTCCGTTCTTCTAAAAAAGGGAAATTGCCTATAGTTGA 910
Pj_impdh_Cisse_CDS      AGGGTATTACCCTTGAAGAGGCAAATGAAATCCTCCGTTCTTCTAAAAAAGGGAAATTGCCTATAGTTGA 671

                           *
Pj_impdh_Ma_genomic     TAAGAATGGGAATCTTACAGCTCTTCTTTCTCGCTCTGATTTAATGAAAAATCTTCATTTCCCATTATCA 980
Pj_impdh_Ma_CDS         TAAGAATGGGAATCTTACAGCTCTTCTTTCTCGCTCTGATTTAATGAAAAATCTTCATTTCCCATTATCA 741
Pj_impdh_Cisse_genomic  TAAGAATGGGAATCTTACAGCTCTTCTTTCTCGCTCTGATTTAATGAAAAATCTTCATTTCCCATTATCA 980
Pj_impdh_Cisse_CDS      TAAGAATGGGAATCTTACAGCTCTTCTTTCTCGCTCTGATTTAATGAAAAATCTTCATTTCCCATTATCA 741

                                                               *
Pj_impdh_Ma_genomic     TCTAAATTACCCGACTCTAAACAATTAATTTGTGCAGCAGCAGTTGGAACACGACCTGAAGATAGAATTA 1050
Pj_impdh_Ma_CDS         TCTAAATTACCCGACTCTAAACAATTAATTTGTGCAGCAGCAGTTGGAACACGACCTGAAGATAGAATTA 811
Pj_impdh_Cisse_genomic  TCTAAATTACCCGACTCTAAACAATTAATTTGTGCAGCAGCAGTTGGAACACGACCTGAAGATAGAATTA 1050
Pj_impdh_Cisse_CDS      TCTAAATTACCCGACTCTAAACAATTAATTTGTGCAGCAGCAGTTGGAACACGACCTGAAGATAGAATTA 811
```

```
Pj_impdh_Ma_genomic      GATTAAAATATTTAGTTGACGCGGGATTAGACATTGTTGTTCTAGATTCAAGTCAGGGGAATTCTATCTA 1120
Pj_impdh_Ma_CDS          GATTAAAATATTTAGTTGACGCGGGATTAGACATTGTTGTTCTAGATTCAAGTCAGGGGAATTCTATCTA 881
Pj_impdh_Cisse_genomic   GATTAAAATATTTAGTTGACGCGGGATTAGACATTGTTGTTCTAGATTCAAGTCAGGGGAATTCTATCTA 1120
Pj_impdh_Cisse_CDS       GATTAAAATATTTAGTTGACGCGGGATTAGACATTGTTGTTCTAGATTCAAGTCAGGGGAATTCTATCTA 881

Pj_impdh_Ma_genomic      TCAAATTAATATGATTAAATGGATAAAAAAGGAATTTCCTGGTTTAGAAATTATAGCTGGAAACGTTGTA 1190
Pj_impdh_Ma_CDS          TCAAATTAATATGATTAAATGGATAAAAAAGGAATTTCCTGGTTTAGAAATTATAGCTGGAAACGTTGTA 951
Pj_impdh_Cisse_genomic   TCAAATTAATATGATTAAATGGATAAAAAAGGAATTTCCTGGTTTAGAAATTATAGCTGGAAACGTTGTA 1190
Pj_impdh_Cisse_CDS       TCAAATTAATATGATTAAATGGATAAAAAAGGAATTTCCTGGTTTAGAAATTATAGCTGGAAACGTTGTA 951

Pj_impdh_Ma_genomic      ACTAGAGAACAAGCTGCTAATTTAATATCAGCTGGAGCAGACGCATTGCGAATAGGTATGGGATCAGGGT 1260
Pj_impdh_Ma_CDS          ACTAGAGAACAAGCTGCTAATTTAATATCAGCTGGAGCAGACGCATTGCGAATAGGTATGGGATCAGG-- 1019
Pj_impdh_Cisse_genomic   ACTAGAGAACAAGCTGCTAATTTAATATCAGCTGGAGCAGACGCATTGCGAATAGGTATGGGATCAGGGT 1260
Pj_impdh_Cisse_CDS       ACTAGAGAACAAGCTGCTAATTTAATATCAGCTGGAGCAGACGCATTGCGAATAGGTATGGGATCAGG-- 1019

                                 *                                                          *
Pj_impdh_Ma_genomic      ATATTAATCTATATTTTTTGAGCTTATAAATTAATATTGTCTAGTTCAATTTGTATAACACAGTAATAAT 1330
Pj_impdh_Ma_CDS          ------------------------------------------TTCAATTTGTATAACACA-------- 1037
Pj_impdh_Cisse_genomic   ATATTAATCTATATTTTTTGAGCTTATAAATTAATATTGTCTAGTTCAATTTGTATAACACAGTAATAAT 1330
Pj_impdh_Cisse_CDS       ------------------------------------------TTCAATTTGTATAACACA-------- 1037

Pj_impdh_Ma_genomic      TTTTTTCGTTTAATTATTAAAATTGATTTTTTGTAGAGAAGTAATGGCAGTTGGGCGGCCTCAAGTATTT 1400
Pj_impdh_Ma_CDS          ------------------------------------AGAAGTAATGGCAGTTGGGCGGCCTCAAG----- 1066
Pj_impdh_Cisse_genomic   TTTTTTCGTTTAATTATTAAAATTGATTTTTTGTAGAGAAGTAATGGCAGTTGGGCGGCCTCAAGTATTT 1400
Pj_impdh_Cisse_CDS       ------------------------------------AGAAGTAATGGCAGTTGGGCGGCCTCAAG----- 1066

Pj_impdh_Ma_genomic      ATTACTTAATAAGTAGATATTATTACTAATTTCAATATTATAGGCAACAGCTGTTTATGCTGTATCAGAA 1470
Pj_impdh_Ma_CDS          ------------------------------------------CAACAGCTGTTTATGCTGTATCAGAA 1092
Pj_impdh_Cisse_genomic   ATTACTTAATAAGTAGATATTATTACTAATTTCAATATTATAGGCAACAGCTGTTTATGCTGTATCAGAA 1470
Pj_impdh_Cisse_CDS       ------------------------------------------CAACAGCTGTTTATGCTGTATCAGAA 1092

Pj_impdh_Ma_genomic      TTTGCATCTAAATTCGGAGTACCAACTATTGCAGATGGAGGAATCGAAAACATTGGGTAAATAAAAACAT 1540
Pj_impdh_Ma_CDS          TTTGCATCTAAATTCGGAGTACCAACTATTGCAGATGGAGGAATCGAAAACATTG--------------- 1147
Pj_impdh_Cisse_genomic   TTTGCATCTAAATTCGGAGTACCAACTATTGCAGATGGAGGAATCGAAAACATTGGGTAAATAAAAACAT 1540
Pj_impdh_Cisse_CDS       TTTGCATCTAAATTCGGAGTACCAACTATTGCAGATGGAGGAATCGAAAACATTG--------------- 1147

Pj_impdh_Ma_genomic      AATATATAATTCATAGATTTATCTAAATAGTCATATAACAAAAGCACTAGCATTAGGAGCATCTGCTGTT 1610
Pj_impdh_Ma_CDS          -----------------------------GTCATATAACAAAAGCACTAGCATTAGGAGCATCTGCTGTT 1188
Pj_impdh_Cisse_genomic   AATATATAATTCATAGATTTATCTAAATAGTCATATAACAAAAGCACTAGCATTAGGAGCATCTGCTGTT 1610
Pj_impdh_Cisse_CDS       -----------------------------GTCATATAACAAAAGCACTAGCATTAGGAGCATCTGCTGTT 1188

Pj_impdh_Ma_genomic      ATGATGGGCAATCTTTTAGCTGGAACAGCAGAATCTCCTGGTCAATATTATTACAGAGATGGTCAACGTT 1680
Pj_impdh_Ma_CDS          ATGATGGGCAATCTTTTAGCTGGAACAGCAGAATCTCCTGGTCAATATTATTACAGAGATGGTCAACGTT 1258
Pj_impdh_Cisse_genomic   ATGATGGGCAATCTTTTAGCTGGAACAGCAGAATCTCCTGGTCAATATTATTACAGAGATGGTCAACGTT 1680
Pj_impdh_Cisse_CDS       ATGATGGGCAATCTTTTAGCTGGAACAGCAGAATCTCCTGGTCAATATTATTACAGAGATGGTCAACGTT 1258

                                                                                      *
Pj_impdh_Ma_genomic      TAAAATCATATCGTGGAATGGGCTCCATTGATGCTATGGAACATTTATCGGGAAAAGGCAAAGGAGAAAA 1750
Pj_impdh_Ma_CDS          TAAAATCATATCGTGGAATGGGCTCCATTGATGCTATGGAACATTTATCGGGAAAAGGCAAAGGAGAAAA 1328
Pj_impdh_Cisse_genomic   TAAAATCATATCGTGGAATGGGCTCCATTGATGCTATGGAACATTTATCGGGAAAAGGCAAAGGAGAAAA 1750
Pj_impdh_Cisse_CDS       TAAAATCATATCGTGGAATGGGCTCCATTGATGCTATGGAACATTTATCGGGAAAAGGCAAAGGAGAAAA 1328

                            *
Pj_impdh_Ma_genomic      TGCCGCATCTAGTCGTTATTTTGGTGAAACAGATACTATTCGTGTTGCCCAAGGTGTTTCAGGAAGTGTT 1820
Pj_impdh_Ma_CDS          TGCCGCATCTAGTCGTTATTTTGGTGAAACAGATACTATTCGTGTTGCCCAAGGTGTTTCAGGAAGTGTT 1398
Pj_impdh_Cisse_genomic   TGCCGCATCTAGTCGTTATTTTGGTGAAACAGATACTATTCGTGTTGCCCAAGGTGTTTCAGGAAGTGTT 1820
Pj_impdh_Cisse_CDS       TGCCGCATCTAGTCGTTATTTTGGTGAAACAGATACTATTCGTGTTGCCCAAGGTGTTTCAGGAAGTGTT 1398

                                                    *
Pj_impdh_Ma_genomic      GTGGATAAAGGATCATTACATGTATATCTTCCTTATTTACGAACCGGGCTTCAACATTCATTACAAGATA 1890
Pj_impdh_Ma_CDS          GTGGATAAAGGATCATTACATGTATATCTTCCTTATTTACGAACCGGGCTTCAACATTCATTACAAGATA 1468
Pj_impdh_Cisse_genomic   GTGGATAAAGGATCATTACATGTATATCTCCCTTATTTACGAACCGGGCTTCAACATTCATTACAAGATA 1890
Pj_impdh_Cisse_CDS       GTGGATAAAGGATCATTACATGTATATCTCCCTTATTTACGAACCGGGCTTCAACATTCATTACAAGATA 1468

                                                                                          *
Pj_impdh_Ma_genomic      TCGGTGTACGAAATTTAACGGAACTAAGAAGACAGGTACGGAAAAAAAATGTACGTTTTGAGCTTCGGAC 1960
Pj_impdh_Ma_CDS          TCGGTGTACGAAATTTAACGGAACTAAGAAGACAGGTACGGAAAAAAAATGTACGTTTTGAGCTTCGGAC 1538
Pj_impdh_Cisse_genomic   TCGGTGTACGAAATTTAACGGAACTAAGAAGACAGGTACGGAAAAAAAATGTACGTTTTGAACTTCGGAC 1960
Pj_impdh_Cisse_CDS       TCGGTGTACGAAATTTAACGGAACTAAGAAGACAGGTACGGAAAAAAAATGTACGTTTTGAACTTCGGAC 1538

Pj_impdh_Ma_genomic      TGTTGCAAGTCAATTAGAAGGGAATGTCCACGGTATTTATCAACTTTTAATTAAACCTAATTTAATTTAT 2030
Pj_impdh_Ma_CDS          TGTTGCAAGTCAATTAGAAGGGAATGTCCACGG------------------------------------- 1571
Pj_impdh_Cisse_genomic   TGTTGCAAGTCAATTAGAAGGGAATGTCCACGGTATTTATCAACTTTTAATTAAACCTAATTTAATTTAT 2030
Pj_impdh_Cisse_CDS       TGTTGCAAGTCAATTAGAAGGGAATGTCCACGG------------------------------------- 1571

Pj_impdh_Ma_genomic      ACTAGGGCTGCATTCTTATAAAAAGAAACTCTGGAGTTGA 2070
Pj_impdh_Ma_CDS          ------GCTGCATTCTTATAAAAAGAAACTCTGGAGTTGA 1605
Pj_impdh_Cisse_genomic   ACTAGGGCTGCATTCTTATAAAAAGAAACTCTGGAGTTGA 2070
Pj_impdh_Cisse_CDS       ------GCTGCATTCTTATAAAAAGAAACTCTGGAGTTGA 1605
```
