# Supplementary material for: Selection of Pneumocystis jirovecii Inosine 5′-Monophosphate Dehydrogenase Mutants in Solid Organ Transplant Recipients: Implication of Mycophenolic Acid
Source: J Fungi (Basel). 2021 Oct 10;7(10):849. doi: 10.3390/jof7100849 (PMC8537117; doi:10.3390/jof7100849)

Figure S3. Diversity of the *impdh* gene of *Pneumocystis jirovecii* based on the analysis of 26 specimens.

Only the surrounding regions of polymorphic positions are shown. The height of amino acid symbols indicates the relative frequency of each amino acid at that position.

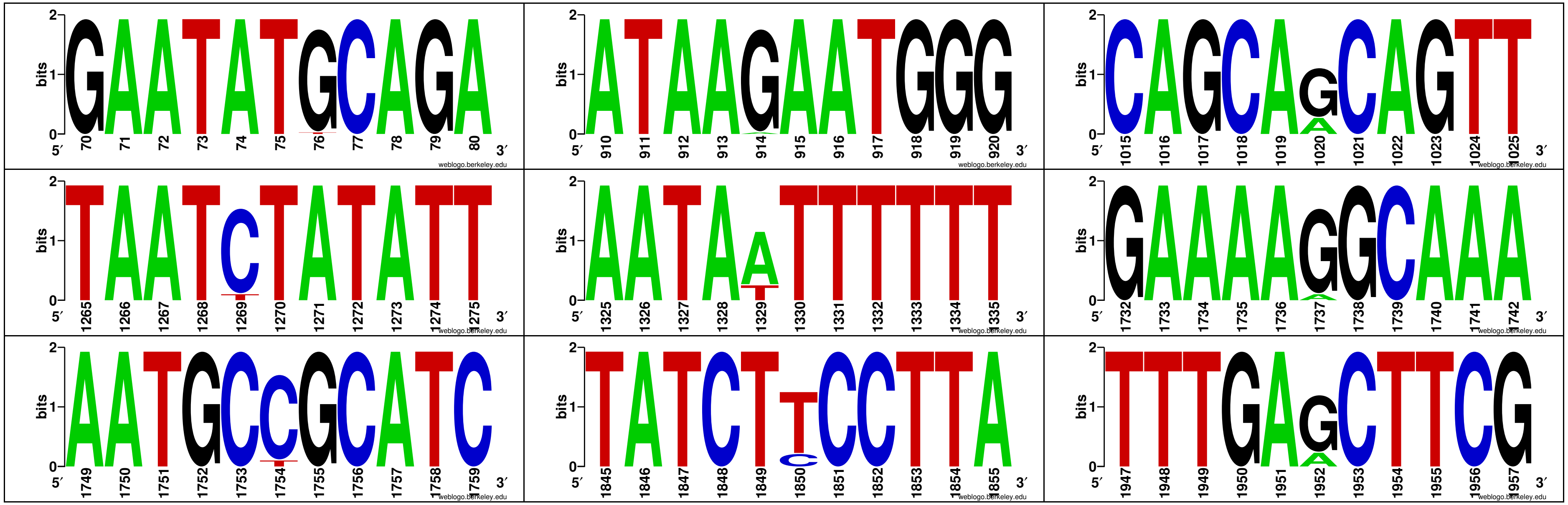

Supplement: Supplementary file 1 [file jof-07-00849-s001.zip › jof-1402122-supplementary/Figure S3.pdf]
